# A Survey of Musculoskeletal Disorders in the Orthopaedic Surgeon: Identifying Injuries, Exacerbating Workplace Factors, and Treatment Patterns in the Orthopaedic Community

**DOI:** 10.5435/JAAOSGlobal-D-20-00244

**Published:** 2022-05-24

**Authors:** Katherine R. Swank, Jamie E. Furness, Erin Baker, Corinn K. Gehrke, Rachel Rohde

**Affiliations:** From the Department of Orthopaedic Surgery (Dr. Swank, Dr. Furness, Dr. Rohde); the Department of Orthopaedic Research (Baker, Dr. Gehrke), Beaumont Health, Royal Oak, MI; and the Department of Orthopaedic Surgery (Baker, Dr. Rohde), Oakland University-William Beaumont School of Medicine, Rochester, MI.

## Abstract

**Introduction:**

As demand for orthopaedic care increases, the orthopaedic community must preserve access to skilled physicians. Workplace hazards and related injuries or conditions contribute to musculoskeletal (MSK) stress on orthopaedic surgeons, which can lead to undesirable medical leaves of absence or early retirement. The purpose of this study was to identify and characterize work-related and non–work-related MSK conditions that affect orthopaedic surgeons and differential injury patterns among male and female surgeons. This study hypothesized that MSK conditions would be exacerbated by work, correlate with age, and show gender-based disparities. Identifying MSK conditions and associated workplace hazards may ultimately help guide preventive or protective efforts.

**Methods:**

Following IRB and society approvals, a modified 15-question physical discomfort survey was emailed to a randomized selection of American Academy of Orthopaedic Surgeons (AAOS) members and all Ruth Jackson Orthopaedic Society members. Data were deidentified and merged by AAOS; analyses were performed by the authors.

**Results:**

Most surgeons reported at least one MSK condition (86%; 95% male versus 82% female, *P* = 0.317), with an average of two conditions per surgeon. Low back pain (56%) and neck pain (42%) were the two most common conditions reported. Male surgeons were more likely to report medial epicondylitis (*P* = 0.040), lateral epicondylitis (*P* ≤ 0.001), low back pain (*P* = 0.001), and lumbar radiculopathy (*P* = 0.001); however, male respondents were significantly older than female respondents (57 versus 43 years, *P* ≤ 0.0001), and some conditions were age-correlated. Most respondents reported at least one work-attributed MSK condition (64%; 68% male versus 62% female, *P* = 0.806). Caseload was not associated with an increased number of work-related MSK conditions; yet, 60% of surgeons reported that work worsened symptoms. Surgical treatment was sought most often for lumbar radiculopathy (6%) and carpal tunnel syndrome (6%). Sixty-nine leaves of absence were reported; most less than 1 month (55%). Exacerbating workplace factors included positioning (patient/surgeon), instruments, and personal protective equipment.

**Discussion:**

Work-related MSK conditions are common among orthopaedic surgeons. Greater awareness of potential workplace-related hazards and conditions is needed to address and mitigate negative MSK health effects on orthopaedic surgeons.

Occupational injuries and hazards have gained increased attention in the surgical community in recent years. Occupational injuries, defined in the United States by the Occupational Safety and Health Administration, include “any wound or damage to the body resulting from an event in the work environment” and are considered to be work-related “if an event or exposure in the work environment either caused or contributed to the resulting condition or significantly aggravated a pre-existing condition.”^1^ Across all sectors, nonfatal occupational injuries cost approximately $190 billion annually, with $46 billion attributable to direct medical costs and $145 billion to loss of productivity.^2^

Radiation, polymethyl methacrylate cement, blood-borne pathogens, anesthetic gases, noise, and physical and psychological/emotional factors resulting in occupational injury have been documented in the setting of orthopaedic surgery.^2^3,4,5^6^ Specifically, spine surgeons have a 25-fold increase in thyroid cancer, likely linked to radiation exposure; polymethyl methacrylate cement has been associated with toxic effects on skin, respiratory, and nervous systems, and smoke inhalation from electrocauterization instruments increases exposure to toxic fumes.^7-9^ Mirbod et al^10^ reported that orthopaedic surgeons have an increased incidence of musculoskeletal (MSK) complaints compared with general surgeons. Factors contributing to increased musculoskeletal system strain and injury of orthopaedic surgeons include repetitive and/or forceful movements, prolonged standing, operating in sustained nonergonomic positions and related muscle fatigue, and poor instrumentation design.^11^12,13^14^ Commonly described work-related musculoskeletal disorders among interventionalists, including orthopaedic surgeons, comprise spinal conditions (eg, degenerative cervical spine disease, neck pain, degenerative lumbar disease, and lumbar pain), shoulder pathology (eg, rotator cuff disease, tendinitis, and impingement), lateral epicondylitis, wrist or forearm tendinitis, carpal tunnel syndrome, knee arthritis, and plantar fasciitis.^2,15^16,17,18^19^

Because demand for orthopaedic care has been predicted to increase exponentially, the performance of each surgeon must be optimized.^2^ Work-related injuries and/or conditions may lead to missed work days, leaves of absence, work restrictions, surgical treatment, and early retirement, affecting the orthopaedic community's ability to provide access to care. Economically, physician injuries and/or conditions may affect not only the individual but also the overall healthcare sector (eg, hospitals and ambulatory surgery centers) because of missed workdays and inability to perform some/all procedures. Additional understanding of the musculoskeletal conditions affecting the orthopaedic surgery community, the effect of these conditions on surgeons' practices, and the specific causes of these conditions may provide a framework to guide the development of preventive efforts, in a time during which orthopaedic surgeon workforce shortages are already anticipated.^20,21^

In this study, work-related and non–work-related MSK conditions that affect orthopaedic surgeons were identified and characterized through a survey of two orthopaedic surgery professional organizations. In addition, differential injury patterns among male and female surgeons were assessed. This study hypothesized that MSK conditions would be exacerbated by work, correlate with age, and show gender-based disparities. Through the identification of MSK conditions associated with workplace-related hazards (eg, instruments and activities), preventive or protective models may be developed and implemented.

## Methods

### Survey Design and Distribution

After obtaining Institutional Review Board, AAOS Women's Health Issues Advisory Board (WHIAB), and Ruth Jackson Orthopaedic Society (RJOS) Executive Board approvals, a 15-question–modified physical discomfort survey was sent through e-mail to a randomized selection of American Academy of Orthopaedic Surgeons (AAOS) and all active, candidate, and resident RJOS members. The survey included demographics-based questions (eg, age, sex, hand-dominance, geographic location, specialty, and years in practice) and musculoskeletal injury–related questions (eg, condition, attribution to work, and leave(s) of absence) (**Supplemental Table 1**, http://links.lww.com/JG9/A214). Leave of absence was defined as any amount of time off from work; additionally, survey respondents were asked to specify the duration of any reported leave of absence, specifically no leave of absence, less than 1 month, 1 to 3 months, 3 to 6 months, 6 months-1 year, greater than 1 year, or permanent leave. Survey data were deidentified and merged by the Department of Research and Scientific Affairs at the American Academy of Orthopaedic Surgeons; subsequently, data and statistical analyses were performed by the authors.

### Statistical Analysis

Data were statistically analyzed (SPSS v.26, IBM), with statistical significance set at α = 0.05 for all tests. Subgroup analyses were performed to evaluate differences between male and female orthopaedic surgeons. Analysis of variance modeling was used to compare continuous data (normality tested with the Shapiro-Wilk test and Kruskal-Wallis analysis of variance used when data were not normally distributed), with a Dunn post hoc test used in multiple comparisons. Fisher exact tests were used to compare categorical variables. Spearman rank order correlations were used to assess notable relationships between clinical and PJI algorithm data; correlation coefficients (ρ) of 0.200 to 0.400, 0.400 to 0.600, 0.600 to 0.800, and 0.800 to 1.000 were considered weak, moderate, strong, and very strong, respectively.^22,23^

## Results

### Demographics of Survey Respondents

A total of 235 orthopaedic surgeons completed the survey during the period of data collection, with a survey response rate of 16%. Respondents included surgeons in various career stages (eg, trainee/resident/fellow, part-time or full-time, or retired) from 44 states in the United States and five countries (**Supplemental Table 2**, http://links.lww.com/JG9/A215); all orthopaedic subspecialties were represented (Table 1). Orthopaedic surgeons in all stages of their careers were included in the survey to allow for characterization of age-related, caseload-related, and work-related MSK injuries. Respondents self-identified sex as female (n = 159) or male (n = 76) surgeons (Table 2). The mean age of the respondents was 47 years (range, 24–81); the mean ages of male and female surgeons were 57 years (range, 36–81) and 43 years (range, 24–68), respectively, which was significantly different between these groups (*P* ≤ 0.0001). The mean length of practice was 20 years (range, 0–53), with male respondents in practice significantly longer than female surgeons (*P* ≤ 0.0001; male: 29 years, female: 15 years). Self-reported surgical time averaged 15 hours per week (range, 1–60; male: 16 hours per week, female: 15 hours per week) while reported clinic time was 23 hours per week (range, 2–60; male: 24 hours per week, female: 22 hours per week); neither case nor clinic hours were significantly different between male and female surgeons (*P* =0.785 and *P* = 0.214, respectively).

**Table 1:** Distribution of Orthopaedic Subspecialties of Survey Respondents

Specialty	Total	Male	Female	*P*
Adult hip	1	1	0	N/A
Adult knee	7	6	1	N/A
Adult spine	5	4	1	N/A
Arthroscopy	4	4	0	N/A
Disability/legal orthopaedics	1	0	1	N/A
Foot and ankle	13	4	9	N/A
Hand	33	8	25	0.005
Nonsurgical	1	1	0	N/A
Orthopaedic oncology	9	2	7	N/A
Pediatric orthopaedics	30	4	26	N/A
Pediatric spine	0	0	0	N/A
Rehabilitation^a^	1	0	1	N/A
Shoulder and elbow	9	3	6	N/A
Sports medicine	27	9	18	0.637
Total joints	23	12	11	0.779
Trauma	12	6	6	0.394
No specialty/general	38	10	28	0.056
Others/trainee^b^	21	2	19	N/A

a Includes prosthetics and orthotics.

b Respondents were trainees or did not enter a specialty and were included in “others”.

**Table 2:** Demographic and Practice-Based Information of Respondents^a^

Variable	Total	Male	Female	*P*
Age (yr; average)	47 (range, 24-81)	57 (range, 36-81)	43 (range, 24-68)	≤0.0001
Sex (frequency)	235	76 (32%)	159 (68%)	N/A
Years in practice (average)	20 (range, 0-53)	29 (range, 10-53)	15 (range, 0-41)	≤0.0001
Surgery hours/week (average)	15 (range, 1-60)	16 (range, 4-42)	15 (range, 1-60)	0.785
Office hours/week	23 (range, 2-60)	24 (range, 5-44)	22 (range, 2-60)	0.214

a Additional demographic information is available in Table 1 (subspecialty distribution) and **Supplemental Table 2** (geographic distribution).

### Self-Reported Musculoskeletal Conditions

Most respondents reported experiencing symptoms of at least one musculoskeletal condition since beginning work as an orthopaedic surgeon (total: n = 202, 86%; male: n = 72, 95%; female: n = 130, 82%). The most commonly reported condition was low back pain (56%), followed by neck pain (42%), rotator cuff tendinitis (33%), carpal tunnel syndrome (33%), others/self-reported (32%), lateral epicondylitis (30%), plantar fasciitis (29%), and basilar joint arthritis (28%) (Table 3). Other musculoskeletal conditions reported by less than 30% of respondents included cubital tunnel syndrome, sciatica, lumbar radiculopathy, cervical radiculopathy, trigger finger, biceps tendinitis, DeQuervain tenosynovitis, medial epicondylitis, and acromioclavicular joint arthritis. Proportionally, male surgeons reported greater rates of medial epicondylitis (*P* = 0.040), lateral epicondylitis (*P* ≤ 0.001), low back pain (*P* = 0.001), and lumbar radiculopathy (*P* = 0.001) than female surgeons; however, male respondents were also markedly older than female respondents, and some conditions were weakly-to-moderately correlated with age (Table 4). Lateral epicondylitis, lumbar radiculopathy, and basal joint arthritis had the greatest correlations with age.

**Table 3:** Surveyed Musculoskeletal Conditions

Musculoskeletal Condition	Work-Related		Non–Work-Related		Total Conditions Reported	
	Male	Female	Male	Female	Male	Female
Carpal tunnel syndrome	15	19	14	29	29	48
Trigger finger	7	11	9	10	16	21
Basal joint arthritis	12	31	12	10	24	41
DeQuervain tenosynovitis	8	6	1	14	9	20
Cubital tunnel	3	10	12	36	15	46
Medial epicondylitis	6	2	6	8	12	10
Lateral epicondylitis	19	20	20	12	39	32
Biceps tendinitis	4	6	5	14	9	20
Rotator cuff tendinitis^a^	10	16	24	28	34	44
AC joint arthritis	1	5	7	5	8	10
Cervical radiculopathy	10	15	8	10	18	25
Neck pain	22	37	18	22	40	59
Low back pain	33	43	24	32	57	75
Lumbar radiculopathy	11	10	13	10	24	20
Sciatica	11	13	11	14	22	27
Plantar fasciitis	7	18	18	24	25	42
Others^b^	6	12	20	37	26	49
Total Per sex cohort	185	274	222	315	407	589
Total Per work attribution cohort	459		537		996	

AC = acromioclavicular

a Rotator cuff tendinitis included rotator cuff tears and rotator cuff impingement.

b Others (specify): Conditions attributed to work were extensor carpi ulnaris tendinopathy (n = 3, including instability and rupture), plantar foot callus (n = 1), osteoarthritis of hands (n = 1), periscapular shoulder/upper back pain (n = 1), hip pain (n = 1), blunt trauma (n = 1), ulcer (n = 1), and posterior tibial tendinitis (n = 1); n = 8 conditions were unspecified. Conditions not attributed to work included achilles tendinosis/rupture (n = 4), meniscus tear (n = 3), hip pain (n = 3, including arthritis), bilateral knee osteoarthritis (n = 2, including bilateral), foot/ankle arthritis (n = 2, including subtalar), anterior cruciate ligament tear (n = 2), stress fracture (n = 1), paronychia (n = 1), piriformis syndrome (n = 1), gluteus medius tear (n = 1), navicular osteochrondritis dissecans (n = 1), PIP fracture dislocation (n = 1), second metatarsophalangeal joint synovitis (n = 1), rheumatoid arthritis (n = 1), brachial neuritis (n = 1), neuronal hypersensitivity (n = 1), ganglion cyst (n = 1), flexor tenosynovitis (n = 1), trochanteric bursitis (n = 1), seasamoiditis (n = 1), knee pain (n = 1), and osteoarthritis location unspecified (n = 1); n = 27 conditions were unspecified.

**Table 4:** Correlations Between Musculoskeletal Conditions and Respondent Age

Musculoskeletal Condition	Correlation Coefficient (ρ)	*P*	Strength
Lateral epicondylitis	0.467	≤0.0001	Moderate
Lumbar radiculopathy	0.338	≤0.0001	Weak
Basal joint arthritis	0.300	≤0.0001	Weak
Sciatica	0.282	≤0.0001	Weak
Low back pain	0.273	≤0.0001	Weak
Rotator cuff tendinitis	0.249	≤0.001	Weak
Trigger finger	0.242	≤0.001	Weak
Carpal tunnel syndrome	0.206	0.002	Weak

Each condition was self-reported as work-related or non–work-related. Respondents reported 996 instances of musculoskeletal conditions, including 459 instances (46%) of work-related musculoskeletal conditions and 537 (54%) musculoskeletal conditions not attributed to work. Male and female respondents reported a total of 185 (40%) and 274 (60%) work-related musculoskeletal conditions, respectively, with 151 respondents (64%) reporting at least one work-related musculoskeletal condition (male: n = 52, 68%; female: n = 99, 62%). For non–work-related musculoskeletal conditions, 222 instances (41%) were reported by male surgeons while 315 (59%) were reported by female respondents; in addition, 164 respondents (70%) reported at least one non–work-related musculoskeletal condition (male: n = 57, 75%; female: n = 107, 67%).

**Figure 1:**
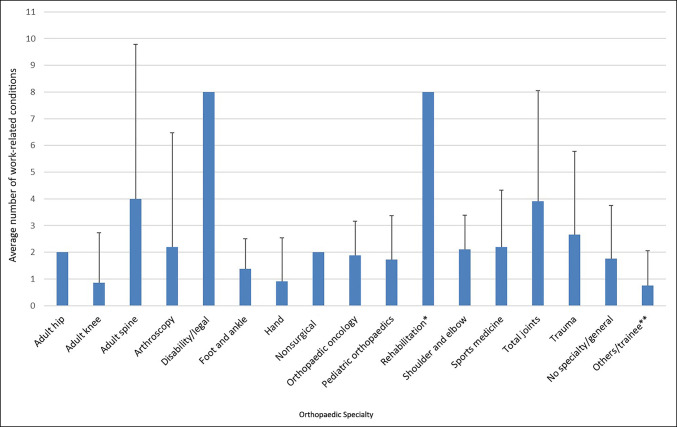
Graph showing average number of work-related musculoskeletal conditions by orthopaedic specialty. *Includes prosthetics and orthotics; **n = 9 female respondents did not enter a specialty and were included in “others.”

Both male and female surgeons averaged two work-related musculoskeletal conditions (male: 2.4; range 0-14; female: 1.7; range, 0–12). When specific work-related conditions were assessed, male surgeons reported significantly greater rates of carpal tunnel syndrome (*P* ≤ 0.001; 20% versus 12%), lateral epicondylitis (*P* ≤ 0.001; 25% versus 13%), rotator cuff tendinitis (*P* ≤ 0.001; 13% versus 10%), low back pain (*P* ≤ 0.001; 43% versus 27%), lumbar radiculopathy (*P* ≤ 0.001; 14% versus 6%), and sciatica (*P* ≤ 0.001; 14% versus 8%). Proportionally, work-related reporting of the 10 other musculoskeletal conditions was not significantly different between male and female respondents. Surgical caseload was not associated with an increased number of self-reported, work-related musculoskeletal conditions among either male (ρ = 0.212, *P* = 0.071) or female surgeons (ρ = 0.018, *P* = 0.828). In male respondents, age (ρ = 0.003, *P* = 0.980) and years in practice (ρ = 0.021, *P* = 0.860) were also not correlated with the number of work-related musculoskeletal conditions; however, in female surgeons, age was weakly correlated with the number of work-related musculoskeletal conditions (ρ = 0.204, *P* = 0.010), but the very weak correlation with years in practice did not meet the reporting threshold defined in the methods (ρ = 0.194, *P* = 0.016). The number of work-related musculoskeletal injuries and/or conditions was also compared with orthopaedic subspecialty, but because of inconsistent distributions between specialties, statistical analysis was performed only for specialties with a minimum of six female and male respondents (hand, sports medicine, total joints, trauma, and no specialty/general; Figure 1). Female hand surgeons reported significantly more work-related musculoskeletal conditions than male hand surgeons (*P* = 0.005). For all other specialties analyzed, no significant differences were observed in the number of work-related musculoskeletal conditions reported by female and male surgeons (sports medicine, *P* = 0.637; total joints, *P* = 0.779; trauma, *P* = 0.394; and no specialty/general, *P* = 0.056) (Table 1).

Respondents also indicated whether their musculoskeletal conditions were exacerbated by their work as orthopaedic surgeons. On average, 60% of surgeons reported that work worsened their musculoskeletal conditions or injuries. Proportionally, of the respondents reporting exacerbations of their conditions, basal joint arthritis (77%), DeQuervain tenosynovitis (72%), cervical radiculopathy (72%), and neck pain (70%) were most commonly affected by work-related activities. Female surgeons reported that work exacerbated basal joint arthritis (*P* = 0.013; 88% versus 67%), cubital tunnel syndrome (*P* = 0.003; 61% versus 27%), lateral epicondylitis (*P* ≤ 0.001; 73% versus 60%), rotator cuff tendinitis (*P* = 0.013; 56% versus 35%), low back pain (*P* = 0.003; 70% versus 66%), lumbar radiculopathy (*P* = 0.003; 70% versus 64%), and plantar fasciitis (*P* ≤ 0.001; 60% versus 44%) at greater rates than male surgeons, whereas male surgeons reported that work-related activities exacerbated neck pain (*P* = 0.003; 72% versus 68%) at greater rates than female surgeons.

### Treatment of Self-Reported Musculoskeletal Conditions

For each musculoskeletal condition, respondents recorded whether treatment was pursued; if so, treatment was further described as observation, nonsurgical, or surgical. Approximately 27% of respondents pursued treatment. On average, among reported musculoskeletal conditions, observation was pursued 14% of the time, 10% pursued nonsurgical treatment, and 3% underwent surgical treatment. Plantar fasciitis (20%), low back pain (19%), and lateral epicondylitis (17%) were reported as the conditions with the greatest rates of nonsurgical treatment while other musculoskeletal conditions (12%; Table 3), lumbar radiculopathy (6%), and carpal tunnel syndrome (6%) had the greatest rates of surgical treatment, with percentages representing the percent of the total percent of each reported musculoskeletal condition. Proportionally, male respondents were less likely than female respondents to pursue treatment for any musculoskeletal condition (*P* = 0.397; 16% versus 13%), nonsurgical treatment (*P* = 0.146, 13% versus 9%), and surgical treatment (*P* = 0.915, 3% versus 2%); however, these results were not statistically significant. Assessing the 16 musculoskeletal conditions defined in the survey, male respondents were significantly more likely to pursue treatment for sciatica compared with female respondents (*P* = 0.001).

Male and female surgeons reported 69 instances of leaves of absences because of musculoskeletal conditions, ranging from less than 1 month to permanent. Other musculoskeletal conditions (n = 19; Table 3), rotator cuff tendinitis (n = 10), and carpal tunnel syndrome (n = 10) were the conditions with the most instances of leaves of absence. Most leaves of absence reported by male surgeons were due to sciatica (n = 6), other musculoskeletal conditions (n = 5), and rotator cuff tendinitis (n = 4) while female surgeons listed other musculoskeletal conditions (n = 14), carpal tunnel syndrome (n = 7), and rotator cuff tendinitis (n = 6). For all respondents, leaves of absence were most commonly less than one month (n = 38; 55%) or 1 to 3 months (n = 26; 38%) in length; however, longer leaves of absence were reported, including 3 to 6 months (n = 2), greater than 1 year (n = 2), and permanent (n = 1) (Table 5).

**Table 5:** Number of Instances of Musculoskeletal Conditions Resulting in Leave of Absence

Leave of Absence	Male	Female	Total
None^a^	171	238	409
<1 mo	14	24	38
1-3 mo	10	14	24
3-6 mo	0	2	2
6-12 mo	0	0	0
>1 yr	0	2	2
Permanent	0	1	1

a No leaves of absence were associated with 409 self-reported conditions.

### Activities and Instruments

Specific activities and instruments that caused or worsened conditions included positioning (both the surgeon and the patient), instruments, personal protective equipment, computer use, long cases, and workplace accidents (Table 6). Positioning of both the surgeon and the patient (ie, lifting, holding, table height limitations, downward gaze, bending, and standing/time on feet) was the primary activity attributed to causing/worsening musculoskeletal conditions (51% of reported instruments or activities). Improperly sized and fitted equipment (eg, loupes and lead aprons) and mismatch between grip size and tool size were reported as exacerbating factors.

**Table 6:** Instruments or Activities Attributed to “Causing” or “Worsening” Musculoskeletal Conditions

Instrument or Activity^a^	Total (%)
Positioning	77 (51)
Instruments	45 (30)
Personal protective equipment	15 (10)
Computer	7 (5)
Length of case	5 (3)
Accident	3 (2)
Total	152

a Instruments or activities defined as: positioning, includes the patient or the surgeon: lifting, holding, table height, neck flexion, bending, and standing/time on feet; instruments: retractors, rongeurs (angled, Kerrison, or pituitary), pickups, hammer/mallet, clamps, screwdriver, drill, reamer, or others (arthroscopy, loupes, gripping/grasping, torsion/twisting, knot typing, knot pusher, shaver, or cautery wand); personal protective equipment: lead aprons and surgical hoods/lights; and accident: blunt trauma by instrument, operating room equipment, or implant

## Discussion

In this series, most surgeons reported at least one MSK condition (86%); however, only 64% of these conditions were attributed to work. Low back pain (56%) and neck pain (42%) were the two most common conditions reported while surgeons pursued surgical treatment most often for lumbar radiculopathy (6%) and carpal tunnel syndrome (6%), resulting in 69 leaves of absence in the series. On average, male respondents were markedly older than female respondents. Caseload (surgery hours/week) and administrative load (office hours/week) were not associated with an increased number of work-related MSK conditions. Approximately 60% of surgeons reported that work worsened symptoms, and self-reported workplace factors that exacerbated MSK conditions included positioning (patient/surgeon), instruments, and personal protective equipment.

Several studies in the literature have identified occupational hazards in orthopaedic surgery, including musculoskeletal injuries and conditions. Davis et al reported that 44% of orthopaedic surgeons had sustained one or more injuries in the workplace while several surveys of orthopaedic subspecialists showed that 62% to 67% of respondents had musculoskeletal injuries, a greater rate than observed in the general population.^16^17,18^19,24^ In this survey, 86% of surgeons reported experiencing at least one musculoskeletal condition since beginning their career, with 64% of respondents attributing at least one musculoskeletal condition to work. Although there may be differences in survey design that would capture a wider variety of musculoskeletal injuries and conditions, reasons for this discrepancy in self-reported prevalence of musculoskeletal injury among orthopaedic surgeons surveyed remains unclear.

As in other studies, low back and neck pain were commonly reported.^16^17,18^19^ Population-based studies of low back pain reported global prevalence of 2.0 to 25.4% while 56% of surgeon respondents (n = 132) reported low back pain, both work-related (n = 76; 32%) and non–work-related (n = 56; 24%).^25^ Neck pain was also common among surveyed orthopaedic surgeons, with 42% of surgeons reporting neck pain (25% work-related and 17% non–work related), which is comparable with a reported prevalence in the general population of 30% to 50% per year.^26^ Prevalence of lumbosacral radiculopathy is reported to be approximately 3% to 5% in the United States, equally distributed among men and women. In this series, 14% of male respondents (n = 11) and 6% of female respondents (n = 10) self-reported lumbar radiculopathy.^27^

Upper extremity conditions were also self-reported at a greater rate in this population than in other studies. In a survey of nearly 10,000 working adults, Walker-Bone et al^28^ found a prevalence of shoulder tendinitis of 4.5% in male and 6.1% in female subjects (versus 33% in this survey), as well as a 1.3% male prevalence and 1.1% female prevalence of lateral epicondylitis (versus 30% in this survey). Symptomatic carpal tunnel syndrome was also more common in this survey than in other population-based studies; specifically, Atroshi et al^29^ reported a 14.4% prevalence of carpal tunnel syndrome symptoms, compared with 33% of self-reporting surgeons in this survey.

Corresponding with the demographics of the 2016 and 2018 AAOS census reports, the surveyed male respondents were markedly older than the female respondents.^30,31^ Like Alzahrani et al^16^ and AlQahtani et al^18^ reported, this survey showed that number of musculoskeletal conditions self-reported was correlated with age and years in practice. For the entire series, respondent sex was not a notable risk factor for work-related injuries and conditions, although female surgeons reported that work was more likely to exacerbate symptoms in select cases (basal joint arthritis, cubital tunnel syndrome, lateral epicondylitis, rotator cuff tendinitis, low back pain, lumbar radiculopathy, and plantar fasciitis). Focusing on subspecialties, only hand surgeons showed notable differences in the number of work-related conditions between female and male hand surgeons; specifically, female hand surgeons reported more work-related conditions. However, a lack of existing research is available to understand this relationship.

Approximately 3% of respondents pursued surgical treatment across all conditions, with highest rates of operation for lumbar radiculopathy and carpal tunnel. Respondents with lumbar radiculopathy and carpal tunnel syndrome diagnoses had the greatest rates of surgical treatment; for each of these conditions, 6% of survey respondents in each group pursued surgical treatment. Farjado et al^32^ reported an incidence in the United States of one to three cases of carpal tunnel syndrome (CTS) per 1,000 patients (0.1% to 0.4%), with an incidence of carpal tunnel release (CTR) of 2/1,000 in men and 4/1,000 in women, indicating that this series of orthopaedic surgeons demonstrated an increased rate of CTS diagnosis and surgical treatment compared with the general population. Because multiple causes of lumbar radiculopathy exist, and thus various surgical treatment options are used based on individual diagnoses, a reliable comparison of specific surgical treatments for this condition could not be performed based on existing survey results of this population. Surgeons were most likely to require leaves of absence because of rotator cuff tendinitis (n = 10) and carpal tunnel syndrome (n = 10).

Numerous respondents reported that specific instruments and activities caused or worsened musculoskeletal injuries or conditions. Specific instruments and activities reported in our study include retractors, Kerrison rongeur, drills, reamers, lead aprons, surgical hoods, loupes, and surgeon or patient positioning. Forst et al^33^ described the association between the use of Kerrison rongeur and increased prevalence of carpal tunnel syndrome in spine surgeons. Catanzarite et al^34^ affirmed the importance of ergonomic intervention as not only beneficial to the individual but also “improving workplace efficiency, reducing cost, decreasing waste of materials and equipment, enhancing corporate image, and increasing employee satisfaction.” Ergonomic hazards in the operating room (eg, inadequate lighting, tripping hazards, improper operating table height, prolonged static positioning, and pressure from instruments) should be mitigated to realize these benefits.^34^ Although some efforts have been made to address these hazards, such as the introduction of microbreak activities and other ergonomic interventions, musculoskeletal injuries remain a notable problem for orthopaedic surgeons.^35,36^ “Physical demands of the field” has been identified as a common deterrent to surgical specialization among medical students, and medical student interest in surgical fields markedly decreased after exposure to literature regarding the incidence of musculoskeletal injuries and conditions among surgeons.^37^ These hazards may also disproportionally affect groups who face additional ergonomic challenges because Rohde et al^38^ identified that a perceived requirement for physical strength may deter women from considering a career in orthopaedic surgery. Although women in surgical specialties are at increased risk of injury requiring treatment for some injury patterns, several studies have reported that employed women are less likely to pursue surgical treatment for work-related injuries.^39-41^ With a projected shortage of 17,100 to 28,700 surgeons by 2033, the importance of recruiting qualified candidates and maintaining the health of the current surgeon workforce should remain a priority.^42^

Limitations do exist with the current study. Because of the survey-based study design, the results rely on the reliability of each subject's ability and desirability to self-report, which may lead to recall, telescoping, and/or social conformity biases that may affect responses. In addition, although respondents attributed musculoskeletal conditions to the workplace, other factors outside the workplace may have also contributed to the onset and exacerbation of the condition. Similarly, injury-exacerbating instruments and activities were submitted in a “free-text” format and were linked inconsistently with a specific condition. Therefore, additional statistical analysis correlation instruments and activities with specific conditions were not possible. In addition, the AAOS census indicates 93.4% and 92.2% male membership in 2016 and 2018, respectively^30,31^; therefore, RJOS members were surveyed to ensure that an adequate number of female surgeons were included to ensure a robust statistical analysis of sex-related differences. Inclusion of both professional societies and varying response rates resulted in male:female distribution that does not accurately reflect the orthopaedic surgeon community (ie, survey = approximately 68% female respondents versus community = approximately 8% female). Intrinsic differences between these two populations (eg, age and training status) may have affected the results of this study. Finally, owing to the number of surgeons surveyed and low response rate, selection bias may disproportionately reflect the experience of surgeons who sustained workplace injuries and conditions.

Work-related musculoskeletal injuries and conditions are common in orthopaedic surgeons because of repetitive and/or forceful movements, prolonged standing, operating in sustained nonergonomic positions, related muscle fatigue, poor instrumentation design, personal protective equipment requirements, and other workplace hazards. Greater awareness of work-related musculoskeletal injuries and conditions that affect orthopaedic surgeons is needed to improve workplace environments. Future studies will aim to determine which instruments and activities are linked with specific musculoskeletal conditions. Knowledge of factors that place orthopaedic surgeons at increased risk may catalyze the development of instruments and techniques to decrease the risk of developing musculoskeletal injuries or minimizing the effects of such conditions on the surgeon, leading to improved health, wellness, and performance capabilities of orthopaedic surgeons.
